# Fortified farm yard manure to bio-stimulate nutrient availability and production of Indian mustard

**DOI:** 10.1371/journal.pone.0342728

**Published:** 2026-05-14

**Authors:** Mukesh Kumar Meena, Ram Swaroop Jat, Mohan Lal Dotaniya, Murli Dhar Meena, Vasudev Meena, Vijay Singh Meena, Ibandalin Mawlong, Ram Lal Choudhary, Harvir Singh, Hari Singh Meena, Vijay Veer Singh

**Affiliations:** 1 ICAR-Indian Institute of Rapeseed-Mustard Research, Bharatpur, India; 2 MJRP College of Agriculture & Research, Mahatma Jyoti Rao Phoole University, Jaipur, India; ICAR-Indian Institute of Pulses Research, APRC, Bikaner, INDIA

## Abstract

Fortified farm yard manure (FYM) enriched with secondary and micronutrients were prepared, and its effects on mitigating the decline in soil health, nutrient availability, and yield levels in Indian mustard under calcareous soil were assessed. FYM at 10 tonnes ha^-1^ and FYM (500 kg ha^-1^) fortified with 20 kg S + 5 kg Zn + 2 kg B + 10 kg Fe significantly improved seed yield and growth parameters of mustard. However, the highest benefit-cost ratio (4.22) with a seed yield of 28.72 q ha^-1^ was obtained under the application of half dose of the recommended levels of secondary and micronutrient, i.e., 2.5 kg Zn + 1 kg B + 5 kg Fe + 10 g S enriched FYM 500 kg ha^-1^. This indicates that application of a balanced amount of micro and secondary nutrients through 500 kg FYM improved mustard yield and economic return to the farmers. Nutrient enrichment with micro and secondary nutrients at the rate of 5.0 kg Zn + 2 kg B + 10 kg Fe + 20 kg S enriched FYM @ 500 kg ha^-1^ significantly increased the soil microbial biomass carbon (56.46% SMB-C), soil organic carbon (SOC) (17.13%), and microbial biomass nitrogen (71.14% MBN) over the control. Moreover, nutrient fortification significantly improved the dehydrogenase activity (56.27%), alkaline phosphatase (101.44%) and aryl sulphatase (73.7%) in soil over control. The availability of S, Zn, Fe, and boron rose by 17.74%, 127.68%, 43.41%, and 52.76%, respectively, compared to the control. The utilization of FYM enriched with secondary and micronutrients at the rate of FYM 500 kg ha^-1^ is an effective technological strategy to improve soil health and production of Indian mustard in semi-arid environments.

## Introduction

With a contribution of over 26.1% to the total oilseed production, mustard is the principal edible oilseed crop in the semi-arid region, following peanut. Raya, or Indian mustard, is regarded as an essential oil-producing crop among Brassica in India [1]. It is a major crop in India’s states of Gujarat, Haryana, Rajasthan, Uttar Pradesh, and Madhya Pradesh [2]. In the area where Indian mustard is grown, the salinity of the soil and water resources poses a major risk. The soils are primarily micronutrient deficient because high saline water irrigation converts available forms of nutrients into forms that are inaccessible to the crop [3]. Crop productivity is low mostly because of abiotic stresses, including soil salinity and sodicity, which limit the availability of micro and secondary nutrients notably, sulphur (S), zinc (Zn), iron (Fe), and boron (B). As a result, careful management of plant nutrients in these soils is just as crucial as soil reclamation. Thus, in nutrient-deficient soil with low carbon content, the yield potential of crops is determined by the application of micro and secondary nutrients [4,5]. The yield of mustard in regular soil was greatly increased by applying micronutrients to the soil in conjunction with FYM [6]. Utilizing FYM and other organic manures causes a variety of organic acids to be produced during the microbial breakdown process, which alter the mobility of plant nutrients from immobile to mobile in the soil solution [7]. Apart from providing micronutrients, organic manures also affect the transformation of native micronutrients in the soil, making them more available to crops [8,9]. Indian mustard exhibits heightened sensitivity to deficiencies in boron (B), nitrogen (N), zinc (Zn), and sulphur (S), leading to reduced crop growth, yield, and productivity [10,11]. High-yielding cultivars are introduced to boost cropping intensity, but restricted additions of organic manure and the use of micronutrient-free fertilizers cause B deficiency in most Indian soils. One of the main factors limiting crop productivity is a lack of boron [12,13]. Due to the intensification of agriculture, there is a daily increase in S deficiency. Soil S supplies are being rapidly depleted due to the use of fertilizer-responsive cultivars. The physico-chemical characteristics and pH of the soil affect the phytoavailability of zinc and iron [14,15]. According to Ghasal et al. [16], 42% of Indian soils now lack enough zinc concentration, and this shortage is predicted to worsen as more marginal lands are put under intense cultivation without receiving enough mineral supplementation. Accordingly, the yield and nutrient content of many oilseed crops have declined due to micronutrient deficiencies in Indian soils, rendering them unfit for human consumption [17]. Most of the knowledge that is currently available on how to optimise the requirements for zinc and iron to compensate for shortages in these minerals in different crops is limited to normal soil conditions. For mustard grown on salt-affected soils in dry and semi-arid locations, such data has not yet been produced. Meena et al. [18] and Singh et al. [19] reported that the incorporation of organic sources into soil as amendments enhances yield by fostering microbial populations and diversity, as well as affecting plant nutrient dynamics. However, a significant issue nowadays is the large-scale availability of FYM. Under saline water irrigation, micronutrient-enriched FYM may therefore be a useful technique for reducing nutrient deficiencies in arid and semi-arid regions. It also aids in lowering the load of chemical fertilizers, lowers soil pH, and aids in the reclamation of alkaline soils. According to Meena et al. [20], the Zn and Fe enriched FYM increased mustard’s uptake of N, S, and micronutrients and enhanced the seed’s oil and protein content. Application of micro and secondary nutrients enriched FYM maintains nutrient availability for an extended period in alkaline ecosystems, perhaps mitigating the detrimental impacts of alkalinity by enhancing soil porosity, infiltration rate, and nutrient mineralization. For oilseed crops to produce more and be of higher quality, the minerals zinc, boron, iron, and sulphur must be supplied and kept in sufficient and accessible proportions. Thus, the goal of the current study was to ascertain how fortified FYM affected Indian mustard output, nutrient concentration and uptake, and nutrient usage efficiency under arid and semi-arid soil conditions.

## Materials and methods

### Site specification and characteristics

The study was carried out during the *Rabi* (winter) season 2016−2021 at the ICAR-Indian Institute of Rapeseed-Mustard Research, Bharatpur, India. The farm is situated at an altitude of 178.37 meters above mean sea level and is located at 77°3’ E longitude and 27°15’ N latitude. The area has a subtropical, semi-arid climatic condition and is located in agro climatic zone IIIa. The procedures outlined by Singh et al. [21] were followed in the collection and analysis of the soil samples. The bulk density of the experimental soil was 1.52 mg m^-3^, its pH (1:2.5) was alkaline in reaction (8.3), and its electric conductivity of saturation extract (ECe) was 1.30dSm^-1^. The texture of the soil was silty clay loam. While being moderate in 0.5N NaHCO_3_ extractable P (17.23 kg ha^-1^) and 1N NH_4_OAc exchangeable K (149.26 kg ha^-1^), the organic carbon (0.24%) and accessible N (126.30 kg ha^-1^) are low. The analysis clearly shows that the soil in the experimental field had an alkaline reaction, was low in organic carbon and phosphorus, had a medium amount of accessible potash, and was low in available nitrogen.

### Treatment details

For Zn, Fe, B, and S enrichment, locally sourced organic materials, such as FYM were utilized. Sixty days before their application in mustard (IJ-31), the enrichment procedure was initiated. A known amount of FYM was filled in the 1.5′ x 1.5′ × 1.5′ pre-dug pits.. Salt solutions of ZnSO_4_.7H_2_O equivalent to 2.5 and 5.0 kilogramme Zn ha^-1^, FeSO_4_.7H_2_O equivalent to 5.0 and 10 kg Fe ha^-1^, Na_2_B_4_O_7_•10H_2_O equivalent to 1.0 and 2 kg B ha^-1^, and SSP and bentonite S corresponding to 10 and 20 kg S ha-1 were delivered using 500 kgha^-1^ well mixed FYM. After mixing with various nutrient sources, the organic material’s moisture percentage was maintained at around 70 percent for the duration of the enrichment process. As a starting inoculum of microorganisms, 1% cow dung slurry was added to the compost to increase the microbiological activities and improve the natural composting process. This process, known as enrichment of FYM, fixed the inorganic Zn, Fe, B, and S that had been added externally into organically bound and naturally chelated forms. A polythene sheet was placed over the pit, and it was left to decay. The periodical samples were taken from the pit for determination of water soluble Zn, Fe, B, and S content, when the value of water soluble nutrients appeared to be more or less constant and the enrichment process was considered as complete. Within six to seven weeks, the process was determined to be nearly finished. Total Zn, Fe, B, and S contents in the FYM employed for enrichment were 66, 1200, 2.4 mg kg^-1^, and 0.24%, respectively (Table 1).

**Table 1:** Nutrient composition of FYM used in the experiment (Mean data of 5 years).

Nutrients	Content
N%	0.54
P%	0.23
K%	0.57
S %	0.24
Fe (mg kg^-1^)	1200.00
Zn (mg kg^-1^)	66.0
B (mg kg^-1^)	2.4

Following enrichment under various levels of micro and secondary nutrients, Table 2 shows the Zn, Fe, B, and S contents in FYM. The Zn, Fe, B, and S-enriched FYM was applied in an experimental field on a pre-selected site, where deficiencies were found in the amount of Zn (0.47 mg kg^-1^ DTPA Zn), Fe (3.6 mg kg^-1^ DTPA Fe), B (0.45 mg kg^-1^ hot water soluble B), and S (8.3 mg kg^-1^turbidimetrically) at the Research Farm. According to Katyal and Rattan [22] and Tandon [23], the soil critical limits for zinc, iron, B, and S are 0.6, 4.0, 0.5, and 10.0 mg kg^-1^, respectively.

**Table 2:** Details of the treatment.

Treatments	Composition
**FYM Level** (Main- plot treatment)	
F_0_ = control	FYM @ 0 t ha^-1^
F_1_ = FYM_5t_	FYM @ 5 t ha^-1^
F_2_ = FYM_10t_	FYM @ 10 t ha^-1^
**Sources of Nutrient** (Sub-plot treatment)	
M_1_ = control	100% Recommended dose of NPK + 500 kg FYM ha^-1^
M_2_ = Zn_1_Fe_1_B_1_S_1_	M_1_ + 2.5 kg Zn + 1 kg B + 5 Kg Fe + 10 kg S ha^-1^
M_3_ = Zn_2_Fe_2_B_2_S_2_	M_1_ + 5 kg Zn + 2 kg B + 10 kg Fe + 20 kg S ha^-1^
M_4_ = En-Zn_1_Fe_1_B_1_S_1_	100% NPK + 2.5 kg Zn + 1 kg B + 5 Kg Fe + 10 kg S Enriched FYM @ 500 kg h^-1^
M_5_ = En-Zn_2_Fe_2_B_2_S_2_	100% NPK + 5 kg Zn + 2 kg B + 10 kg Fe + 20 kg S Enriched FYM @ 500 kg ha^-1^

Studying the effects of nutrient delivery through nutrient-enriched organics and routinely used organics, i.e., FYM without nutrient-enrichment, was done using the recommended dose of NPK (80-40-40) in conjunction with FYM at 500 kg ha^-1^ as a control. This experiment included fifteen treatment combinations (Table 3): three levels of FYM (control (F_0_), FYM @ 5t ha^-1^ (F_1_), and FYM @ 10 t ha^-1^ (F_2_); five enrichment treatments (Zn, Fe, B, and S) at two levels of Zn (2.5 and 5.0 kg Zn ha^-1^) and Fe (5.0 and 10.0 kg Fe ha^-1^) and two levels of S (10 and 20 kg ha^-1^) and B(1 and 2 kg ha^-1^) replicated three times in a split plot design (SPD). A basal dose of urea, SSP, and MOP was administered to each plot, providing half of the recommended nitrogen (80 kg ha^-1^) as well as the entire amount of potassium (40 kg ha^-1^) and phosphorus (40 kg ha^-1^). The Indian mustard variety DRMR IJ-31, the test crop, was cultivated using recommended agronomic practices.

**Table 3:** Total nutrient content of FYM before and after enrichment and nutrient enrichment efficiency.

Organics	En-Zn_1_Fe_1_B_1_S_1_		En-Zn_2_Fe_2_B_2_S_2_		Enrichment efficiency (%)	
Nutrients	Nutrient content before enrichment	Nutrient content after enrichment	Nutrient content before enrichment	Nutrient content after enrichment	En-Zn_1_Fe_1_B_1_S_1_	En-Zn_2_Fe_2_B_2_S_2_
S %	0.24	1.66	0.24	3.39	74.00	79.95
Fe (mg kg^-1^)	1200.00	17534	1200.00	28813	79.70	90.04
Zn (mg kg^-1^)	66.0	3850	66.0	8815	76.00	87.00
B (mg kg^-1^)	2.4	1442	2.4	3082	72.01	77.00

### Soil and plant analysis

Plant samples were taken when the crop reached maturity, and yield data were documented at harvest. After the mustard crop was harvested, soil samples were taken and their nutritional content was examined. Following Jackson’s [24] instructions, the plant materials were subjected to a wet digestion process using a di-acid mixture of HNO_3_: HClO_4_ (4:1). According to Lindsay and Norvell [25], soil samples were extracted using 0.005 M DTPA to assess the micronutrient content, which was then measured using an atomic absorption spectrophotometer. Soil samples were subjected to a conductivity meter analysis for electrical conductivity and a pH meter for pH measurement using a 1:2.5 soil: water solution. Organic carbon estimated by the rapid titration method [26]; available N estimated by the alkaline permanganate method [27]; available P by Olsen’s method [28]; available K by the ammonium acetate extraction method [29]; and available S by the turbidimetric method [30].

### Analysis of soil microbial biomass carbon, nitrogen and enzymatic activities

The chloroform fumigation method measured soil microbial C and N. In this procedure, a 50g soil sample was fumigated for 24 hours under vacuum in a desiccator with ethanol-free chloroform. Soil samples from fumigated and non-fumigated areas were extracted with 0.5 *M* K_2_SO_4_, and the filtrate was used to determine C levels [31], N [32]. The following is a calculation of microbial biomass C, and N,:


$$\begin{array}{c} \text{Microbial biomass carbon }(\text{MBC})=\frac{{OC}_{F}-{OC}_{UF}}{K_{EC}} \end{array}$$ (1)

Where, OC_F_ and OC_UF_ are the organic carbon extracted from fumigated and un-fumigated soil samples, respectively (expressed on the oven-dry basis), and K_EC_ is the efficiency of extraction. A value of 0.45 was considered as a general K_EC_ value for microbial extraction efficiency and is used for calculation.


$$\begin{array}{c} \text{Soil microbial biomass N }=\;\frac{F_{n}}{\text{0}.\text{6}\text{8}} \end{array}$$ (2)

Where, F_n_ = (flush of mineral N in fumigated soil- non- fumigated soil)

For dehydrogenase activity (DHA) estimation, 2, 3, 5- triphenyl tetrazolium chloride (TTC) was used as substrate and measured the formation of triphenyl formazan (TPF) using a spectrophotometer at 485 nm [33]. For alkaline phosphatase activity (APA) estimation, a buffer (pH 9.4) of borax-NaOH and a substrate of p-nitrophenyl phosphate disodium salt were used [34]. Arylsulfatase activity (Arylsul) was determined as described by Alef and Nannipieri [35].

### Estimation of fractions of available nitrogen (NH_4_^+^-N and NO_3_^-^-N)

Soil was extracted with 100 ml of 0.5 M K_2_SO_4_ (shaking for 1 hr) and the amount of NH_4_^+^ and NO_3_^-^ nitrogen was determined by steam distillation method [36].

### Yield and uptake analysis

The net plot area, leaving the boundary rows, was used to measure the yield of seeds and stover, and the results were translated to kg ha^-1^. The samples were air-dried for 48 hours at 65°C in an oven before being measured for the dry weight of various plant parts. The plant materials were ground into a fine powder using a mechanical grinder. On a heated plate, a 3:1 mixture of di-acid, i.e., HNO_3_ and HClO_4_ acid, was used to digest the ground stover and seed samples, which weighed 1.0 and 0.5 g, respectively [37]. The nutrients in the plant’s digested extracts were tested using standard protocol, and their uptakes in Indian mustard seed and stover (g ha^-1^) were computed using Equation:


$$\begin{array}{c} \text{Nutrient uptake g h}a^{-\text{1}}\;=\text{ Concentration }\left(\text{mg k}g^{-\text{1}}\right)\times \text{ Yield }\left(\text{Kg h}a^{-\text{1}}\right)\text{1}\text{0}\text{0}\text{0} \end{array}$$

### Nutrient use efficiency indices

The following formula was used to compute the mobilization efficiency index (MEI) of N, P, K, S, Zn, Fe, and B [38]:


$$\begin{array}{c} MEI=\frac{\text{Nutrient concentration in seed }(\text{mg}/\text{kg})\;}{\text{Nutrient concentration in straw }(\text{mg}/\text{kg})} \end{array}$$

### Economics

Considering the current rates of fertilizer in rupees at the time of application, the cost of fertilizer in rupees per hectare was computed separately for every treatment used in the experiment. Equations 2 and 3 were used to calculate the gross return, or value of additional output, for Indian mustard throughout the study period. The MSP stands for minimum support price, and the Indian government established it.


$$\begin{array}{c} \text{Gross return }=\text{ Yield }\times \text{ Price of produce} \end{array}$$ (3)

$$\begin{array}{c} \text{Net Return }\left(\text{Rs h}a^{-\text{1}}\right)=\text{ Gross return }(\text{Rs h}a^{-\text{1}})\;-\text{ Cultivation cost }(\text{Rs h}a^{-\text{1}}) \end{array}$$

$$\begin{array}{c} \text{B}:\text{C}=\;\frac{\text{Gross return }(\text{Rs h}a^{-\text{1}})}{\text{Cultivation cost }(\text{Rs h}a^{-\text{1}})} \end{array}$$ (4)

### Statistical Analysis

Data were collected and analysed statistically using analysis of variance (ANOVA) in accordance with the protocol outlined in Gomez and Gomez [39]. This was performed to test the significance of the major effects of treatments on the crop. The F-test was used to determine the least significant difference (LSD) using a probability level of p ≤ 0.05 among the main and sub-plot treatment, which included levels of FYM and nutrient enrichment. Mean values of data were evaluated with the least significant difference (LSD) tests to determine the treatment significance.

## Results and discussion

### Grain and Stover Yield

**Fig 1:**
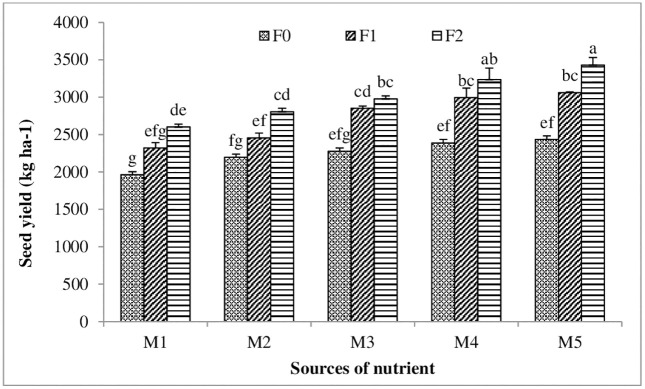
Interactive effects of different level of FYM and sources of nutrient on seed yield of mustard.

The yield data indicated that the application of nutrient-fortified FYM had a major effect on Indian mustard seed and stover yields (Table 4). A mean of 22.95 and 58.14 q ha^-1^, respectively, was noted as the lowest values of seed and stover yield in the control. The treatment with micro and secondary nutrients applied at a rate of 5.0 kg Zn + 2 kg B + 10 kg Fe + 20 g S Enriched FYM @ 500 kg ha^-1^(Zn_2_B_2_Fe_2_S_2_ En) produced the significantly highest seed yield (29.73 q ha^-1^), while the treatment with micro and secondary nutrients applied at a rate of 2.5 kg Zn + 1 kg B + 5 Kg Fe + 10 g S Enriched FYM @ 500 kg ha^-1^(Zn_1_B_1_Fe_1_S_1_En) showed at par effect. When micro and secondary nutrients are added by enriching FYM, the half dose (@5 t ha^-1^) of FYM is adequate to get the significantly largest seed output of mustard. The interactive effect of varied levels of FYM and sources of nutrients on mustard seed yield demonstrated in the Fig 1. The procedure that provided nutrients at the following rates: 5.0 kg Zn + 2 kg B + 10 kg Fe + 20 g S enriched FYM @ 500 kg ha^-1^(Zn_2_B_2_Fe_2_S_2_ En) improved the seed yield by 29.24 and 19.63 percent over control and sole nutrient application at the rate of 2.5 kg Zn + 1 kg B + 5 kg Fe + 10 g S kg ha^-1^(Zn_1_B_1_Fe_1_S_1_), respectively.

**Table 4:** Effect of different level of FYM and sources of nutrient on harvest index and Yield of mustard (Mean data of five years).

Treatments	Seed yield (Kg ha^-1^)	Stover yield (Kg ha^-1^)	Harvest index (%)
**A. FYM Level**			
F_0_ = control	2250.9^c^	6026.1^c^	27.24^b^
F_1_ = FYM_5t_	2736.2^b^	6863.9^b^	28.5^a^
F_2_ = FYM_10t_	3008.8^a^	7567.5^a^	28.44^a^
SEm(±)	39.2	82.3	0.07
LSD (P = 0.05)	154.0	323.1	0.28
**B. Sources of Nutrient**			
M_1_ = control	2295.5^e^	5814.3^e^	28.3^a^
M_2_ = Zn_1_Fe_1_B_1_S_1_	2484.6^d^	6342^d^	28.11^a^
M_3_ = Zn_2_Fe_2_B_2_S_2_	2701.5^c^	6905.6^c^	28.06^a^
M_4_ = En-Zn_1_Fe_1_B_1_S_1_	2871.8^b^	7385^b^	27.92^a^
M_5_ = En-Zn_2_Fe_2_B_2_S_2_	2973.1^a^	7649.1^a^	27.9^a^
SEm(±)	36.0	71.5	0.12
LSD (P = 0.05)	105.0	208.8	0.35

Moreover, the exclusive practice of FYM fortification with micro and secondary nutrients (Zn_1_B_1_Fe_1_S_1_En and Zn_2_B_2_Fe_2_S_2_En) enhanced seed and stover yield by 15.57, 10.03%, and 16.44, 10.13%, respectively, in comparison to the sole application (without fortification with FYM) of nutrients (Zn_1_Fe_1_B_1_S_1_ and Zn_2_Fe_2_B_2_S_2_). Latha et al. [40] have reported on the significance of incorporating micronutrients into FYM to achieve balanced nutrition of various crops and improved yields. Meena et al. [6] have also documented the advantageous impact of FYM enrichment with Zn to boost mustard yield. We found that nutrient fortification of FYM has a significant impact on yield enhancement, and our findings are in close agreement with those of previous studies by Kuppusamy et al. [41], Devarajan and Krishnasamy [42], Meena et al. [20], and Singh et al. [43].

### Soil fertility status after mustard harvest

#### Soil pH, EC and soil organic carbon (SOC).

The application of organic matter in the form of FYM (10 t ha^-1^) reduced soil pH (7.52) when compared to the control (8.04), as the results (Table 5) clearly show. Incorporation of organic manures can help reduce the negative impacts of high pH and sodicity by producing organic acids and CO_2_ through their microbial decomposition. The production of organic acids during the breakdown of organic additions reacts with the soil’s natural CaCO_3_, increasing the amount of calcium in soil solution and substituting Na from the clay complex. This lowers the pH and SAR of the soil [44,45]. Soil EC in all FYM treatments was comparable to the control and did not change substantially. In the instance of OC, rising FYM levels considerably raised the amount of OC in the soil. The OC status of the soil was enhanced from 2.29 to 5.87 g kg^-1^ by applying FYM (10 t ha^-1^). The addition of FYM considerably boosted the OC over the control. The large rise in OC in FYM-treated plots could be attributed to the incorporation of a greater amount of organic material under the treatment of FYM applied at the rate of 10 t ha^-1^. Impact of enriched nutrition of FYM, the pH and EC of the soil were not significantly affected. However, compared to the control (3.56 g kg^-1^), the treatments with micro- and secondary nutrient enriched FYM (Zn_1_B_1_Fe_1_S_1_En and Zn_2_B_2_Fe_2_S_2_ En) increased the soil organic carbon (SOC) by 15.44 and 17.13 percent, respectively. The results of significant improvement in soil organic carbon following the treatment with micro- and secondary-nutrient-supplemented FYM are comparable to those published by Pasricha et al. [46].

**Table 5:** Effect of different level of FYM and sources of nutrient on soil fertility and available nutrient concentration in soil at harvest (Mean data of five years).

Treatments	Available nutrient concentration in soil							Soil chemical properties		
	Zn(mgkg^-1^)	Fe(mg kg^-1^)	B(mg kg^-1^)	S (mg kg^-1^)	N (kg ha^-1^)	P (kg ha^-1^)	K (kg ha^-1^)	pH	EC (dSm^-1^)	SOC (g kg^-1^)
**A. FYM Level**										
F_0_ = control	1.4^a^	8.56^b^	1.41^a^	18.57^c^	118.17^b^	10.09^b^	254.02^b^	8.04^a^	0.57^a^	2.29^c^
F_1_ = FYM_5t_	1.67^a^	9.61^ab^	1.75^a^	24.79^b^	120.94^b^	11.24^ab^	255.81^b^	7.92^a^	0.55^a^	3.35^b^
F_2_ = FYM_10t_	1.78^a^	10.24^a^	1.88^a^	31.16^a^	124.39^a^	12.37^a^	262.54^a^	7.52^a^	0.59^a^	5.87^a^
SEm(±)	0.07	0.20	0.03	0.33	0.93	0.38	1.33	0.04	0.03	0.07
LSD (P = 0.05)	0.29	0.78	0.12	1.29	3.65	1.49	5.21	0.17	NS	0.26
**B. Sources of Nutrient**										
M_1_ = control	0.77^c^	5.85^e^	0.88^d^	20.72^e^	117.15^c^	9.9^b^	236.79^e^	7.82^a^	0.5^a^	3.56^c^
M_2_ = Zn_1_Fe_1_B_1_S_1_	0.92^c^	8.03^d^	1.22 cd	22.22^d^	120.79^b^	10.31^b^	244.12^d^	7.97^a^	0.61^a^	3.65^bc^
M_3_ = Zn_2_Fe_2_B_2_S_2_	1.3^c^	9.18^c^	1.63^bc^	24.7^c^	121.75^b^	10.48^b^	255.9^c^	7.88^a^	0.56^a^	3.69^abc^
M_4_ = En-Zn_1_Fe_1_B_1_S_1_	2.11^b^	11.11^b^	2.17^ab^	27.46^b^	121.33^b^	11.51^b^	269.52^b^	7.66^a^	0.61^a^	4.11^ab^
M_5_ = En-Zn_2_Fe_2_B_2_S_2_	2.97^a^	13.17^a^	2.49^a^	29.09^a^	124.82^a^	13.94^a^	280.94^a^	7.8^a^	0.58^a^	4.17^a^
SEm(±)	0.10	0.20	0.06	0.41	1.45	0.51	1.66	0.10	0.03	0.05
LSD (P = 0.05)	0.29	0.58	0.18	1.21	4.23	1.50	4.84	NS	NS	0.15

### Soil nutrient concentration

The findings (Table 5) showed that higher levels of FYM application are associated with an increase in the available N, P, K, S, and micronutrient (Zn, Fe, and B) content of soil. Following crop harvesting, the maximum availability of these nutrients in the soil was demonstrated under the use of FYM @ 10 t ha^-1^. The direct addition of nutrients brought in the soil on decomposition of organic matter and released organic acids subsequently mobilised natively inaccessible nutrients, which improved the available nutrient status under FYM treatments. Applying organic manures to an alkaline ecosystem can increase soil porosity, infiltration rate, and nutrient mineralization rate, which can mitigate the negative consequences of alkalinity. In addition to providing nutrients that plants need, organic supplementation feeds the soil biota [47,48]. It is also noticed that micro- and secondary-nutrient (Zn, Fe, B and S) -enriched FYM not only influenced the availability of Zn, Fe, B, and S favourably in soil but also improve soil concentration of N, P, and K over the control. It could be the result of improved organic compound mineralisation brought on by organic acids [49]. However, when micro and secondary nutrients were supplied directly (without fortification), the DTPA-extractable levels of Zn, Fe, B, and S in soil solution increased; however, the percentage improvement was less than when the fortified FYM was applied over the control. The creation of stable organometallo complexes between micronutrients and organic matter, particularly during the enrichment process, may also be responsible for the overall increase in micronutrient availability. These complexes last longer and release the nutrients into the soil system gradually, protecting the nutrients from fixation and allowing them to be accessed by the plant root system during crop growth [50]. Chitdeshwari and Krishnasamy [51] conducted a greenhouse experiment to assess the micronutrient concentration in green gram cv. Co 5 and the residual impact of applied zinc and zinc-enriched organic manures. The positive impacts of Zn-enriched organics on soil DTPA-Zn have also been reported. The highest mean Zn levels (3–10 mg/kg in soil and 11.5 mg/kg in leaves) were obtained with the administration of 5.0 mg Zn/kg enriched CCP + GLM. Latha et al. [49] investigated the effects of zinc-enriched organic manures on a maize crop in a field experiment. The effects of organic manures-farmyard manure, chicken manure, coir pith, and biogas slurry enriched with 0, 12.5, and 25.0 kg ZnSO_4_ ha^-1^ on the formation of dry matter, yield, and zinc uptake in maize were assessed. The results showed that using poultry manure increased yield by 26.6% and was superior to using other sources.

### Microbial biomass C (C_mic_), Soil organic carbon (SOC), and microbial quotient (C_mic_/C_org_)

**Fig 2:**
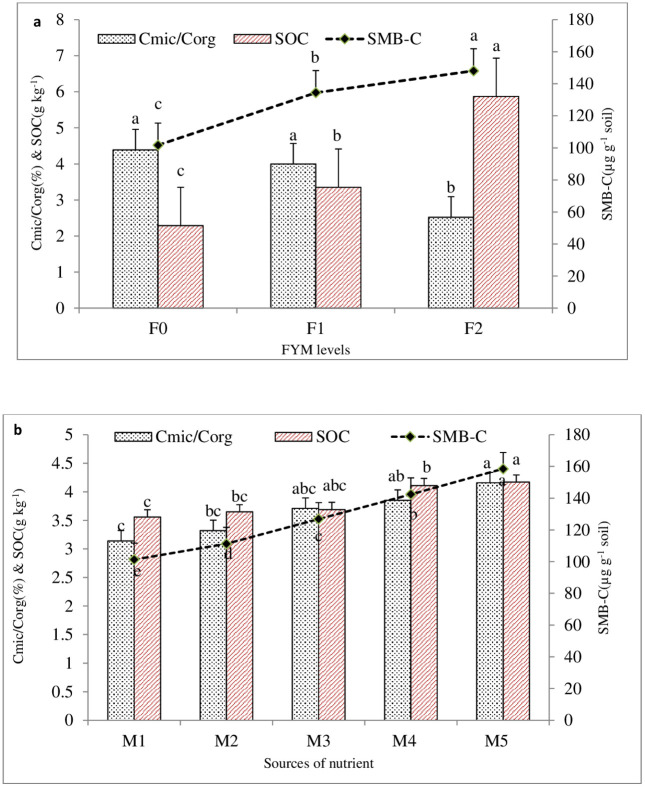
Effect of different level of FYM (a) and sources of nutrient (b) on SOC, SMB-C and C_mic_/C_org_ of soil at harvest.

A five-year period’s time of data on microbial biomass carbon revealed that the addition of organic sources of nutrients (FYM) increased the rate of labile microbial C accumulation significantly (p = 0.05). The buildup rate of C in soil was substantially greater than the control when higher FYM (F2) levels were used (Fig 2a). In comparison to the control (F_0_), the accumulation rate of labile microbial C was increased by 46.28 per cent when 10 t FYM ha^-1^ was added. Meena et al. [48] found a significant relationship between the addition of organic matter to soil and microbial C. The addition of organic matter has been shown by numerous researchers to boost microbial C, N, and P [52,53]. Higher levels of FYM are associated with higher levels of microbial biomass carbon and soil organic carbon (SOC) content, as the data in Fig 2a demonstrate. Increases in microbial biomass carbon and soil organic carbon (SOC) content are also observed upon fortifying FYM with Zn, Fe, B, and S. The straight application of Zn, Fe, B, and S can also (without fortified FYM) increase the microbial biomass carbon content and soil organic carbon (SOC); however the percentage increment was less than when these nutrients were applied through fortified FYM over control (Fig 2b). Higher microbial biomass C, N, and P are the outcome of applying organic sources of nutrients, which also improve soil organic matter [52–55].

Applying higher FYM (F2) levels resulted in a significant (p = 0.05) drop in the microbial quotient, i.e., C_mic_/C_org_, as compared to the control (Fig 2a). Under the treatment of 10 t FYM ha^-1^ incorporated in the field, the C_mic_/C_org_ ratio was much lower and decreased by 42.59% as compared to the control (F_0_). On the other hand, when FYM was fortified with micro- and secondary-nutrients, the C_mic_/C_org_ ratio was significantly higher than it was for the control (Fig 2b). Microbial quotient (C_mic_/C_org_) was enhanced by 22.61 and 32.48 percent over control under the treatment of micro- and secondary-nutrient-enriched FYM (Zn_1_B_1_Fe_1_S_1_En and Zn_2_B_2_Fe_2_S_2_ En) was applied, respectively. The presence of carbon substrate as a source of food materials enhanced microbial growth and enzyme activity, which subsequently declined as the carbon substrate was exhausted, according to Manna et al. [56].

### Microbial biomass N (MBN) and Mineralized fractions of N (NH_4_^+^-N, NO_3_^−^-N)

**Fig 3:**
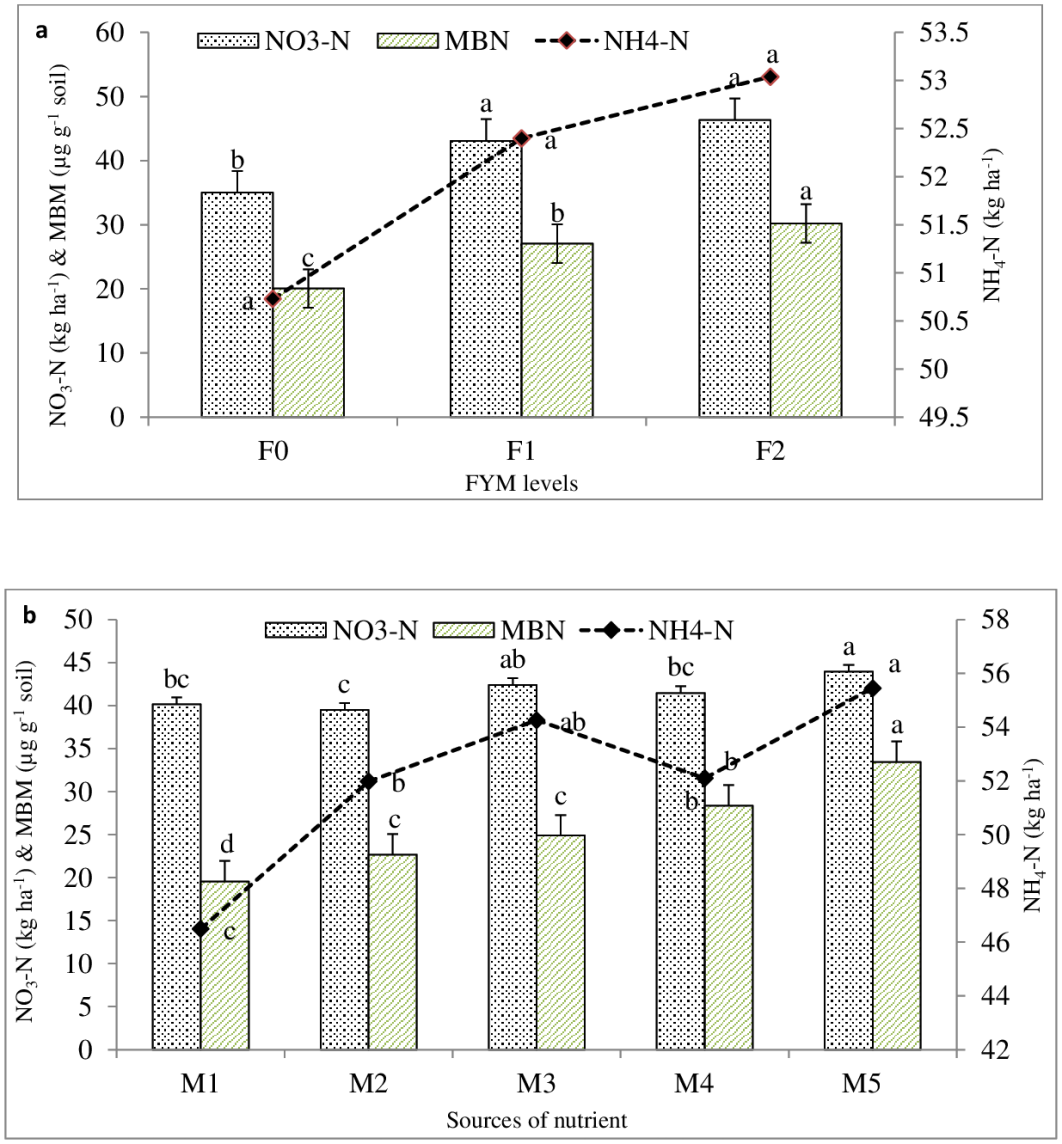
Effect of different levels of FYM (a) and sources of nutrient (b) on soil microbial biomass nitrogen, nitrate nitrogen and ammonical nitrogen of soil at harvest.

With an increase in FYM levels, there was a substantial (p = 0.05) rise in both Microbial Biomass N (MBN) and Soil NO_3_^-^-N contents (Fig 3a). On the other hand, NH_4_^+^-N in the soil showed non- significant improvement. After applying 10 t FYM ha^-1^, the amount of Microbial Biomass N (MBN) and soil NO_3_^-^-N concentrations increased by 50.57 and 32.21%, respectively, compared to the control. The application of organic source nutrients over control resulted in an increase in nitrogen fractions (NH_4_^+^-N and NO_3_^-^-N), and microbial biomass N (MBN) in the soil. This increase may have been caused by the higher mineralization of N from organics in the soil [44,53,57,58].

Increases in microbial biomass N and mineralised components of N (NH_4_^+^-N, NO_3_^-^-N) concentrations in soil are also seen upon enrichment of FYM with Zn, Fe, B, and S (Fig 3b). The straight application (without fortified FYM) of Zn, Fe, B and S also increases microbial biomass N and the mineralised fraction(NH_4_^+^-N, NO_3_^-^-N) of N; however the percentage enhancement was less than when they were applied through the fortified FYM over control. The treatment in which nutrients were applied at the rate of 5.0 kg Zn + 2 kg B + 10 Kg Fe + 20g S enriched FYM @ 500 kg ha-1(Zn_2_B_2_Fe_2_S_2_ En) increased the microbial biomass N and mineralised fractions of N (NO_3_^-^-N, NH_4_^+^-N) contents in soil by 71.13, 9.41 and 19.27% per cent over the control, respectively. The favourable effects of organic manures on the accumulation of exchangeable ammonium nitrogen and the rise in the soil’s organic carbon content may be the cause of increase in ammonium nitrogen. According to Tejada et al. [54,55] and Wu et al. [53], applying Zn, Fe, B, and S-enriched FYM boosts soil organic matter, leading to increased microbial biomass C, N, and P.

### Nutrient content and uptake in seed and stover

Tables 6 and 7 give the findings of the N, P, K, S, Zn, Fe, and B content and uptake in mustard seed and stover as altered by FYM and FYM fortification with S, Zn, Fe, and B levels. A statistical analysis of the data on the concentrations of N, P, K, S, Zn, Fe, and B in seed and stover revealed that these concentrations significantly improved when higher quantities of FYM were applied. It has been noted that mustard seeds have a higher nutrient content than stover, with the exception of K content, which is higher in stover. Furthermore, enriched FYM, including micro- and secondary nutrients (Zn, Fe, B, and S), was seen to improve the concentration of N, P, and K in seed and stover compared to the control, in addition to increasing the content of Zn, Fe, B, and S in seed and stover. Higher amounts of FYM application resulted in a considerable increase in the uptake of N, P, K, S, Zn, Fe, and B in the seed and stover. With all the S, Zn, Fe, and B treatments over control, the removal of N, P, K, S, Zn, Fe, and B in seed and stover was noticeably higher.

**Table 6:** Effect of different level of FYM and sources of nutrient on nutrient content (Seed and Stover) in mustard crop (Mean data of five years).

Treatments	Seed concentration							Stover concentration						
	Zn (mg kg^-1^)	Fe (mg kg^-1^	B (mg kg^-1^)	S (%)	N (%)	P (%)	K (%)	Zn (mg kg^-1^)	Fe (mg kg^-1^	B (mg kg^-1^)	S (%)	N (%)	P (%)	K (%)
**A. FYM Level**														
F_0_ = control	34.07^c^	77.82^b^	23.52^c^	0.368^a^	3.12^a^	0.596^a^	0.415^a^	14.71^c^	62.3^a^	17.89^c^	0.268^a^	0.416^a^	0.200^a^	0.888^a^
F_1_ = FYM_5t_	40.36^b^	80.99^a^	28.63^b^	0.448^a^	3.29^a^	0.612^a^	0.459^a^	16.17^b^	63.16^a^	21.77^b^	0.312^a^	0.43^a^	0.197^a^	0.962^a^
F_2_ = FYM_10t_	45.91^a^	82.79^a^	41.79^a^	0.504^a^	3.44^a^	0.663^a^	0.575^a^	18.57^a^	64.93^a^	29.93^a^	0.333^a^	0.445^a^	0.205^a^	1.143^a^
SEm(±)	0.39	0.76	0.65	0.005	0.01	0.005	0.004	0.13	0.66	0.44	0.003	0.002	0.002	0.008
LSD (P = 0.05)	1.54	3.00	2.54	0.020	0.04	0.020	0.016	0.53	2.59	1.72	0.014	0.008	0.009	0.032
**B. Sources of Nutrient**														
M_1_ = control	28.46^e^	66.04^e^	20.67^e^	0.241^d^	2.59^d^	0.507^c^	0.329^c^	12.84^e^	56.48^d^	18.23^e^	0.21^b^	0.365^a^	0.175^a^	0.887^b^
M_2_ = Zn_1_Fe_1_B_1_S_1_	34.56^d^	73.01^d^	25.72^d^	0.331 cd	2.95^c^	0.567^bc^	0.411^bc^	14.74^d^	61.56^c^	20.56^d^	0.261^ab^	0.401^a^	0.190^a^	0.938^ab^
M_3_ = Zn_2_Fe_2_B_2_S_2_	39.65^c^	80.67^c^	30.98^c^	0.423^bc^	3.31^b^	0.618^abc^	0.491^abc^	16.36^c^	64.15^b^	23.09^c^	0.304^ab^	0.436^a^	0.200^a^	0.983^ab^
M_4_ = En-Zn_1_Fe_1_B_1_S_1_	45.72^b^	87.99^b^	36.46^b^	0.545^ab^	3.64^a^	0.685^ab^	0.556^ab^	18.24^b^	67.09^a^	25.58^b^	0.35ab	0.463^a^	0.213^a^	1.048^ab^
M_5_ = En-Zn_2_Fe_2_B_2_S_2_	52.17^a^	94.95^a^	42.73^a^	0.659^a^	3.92^a^	0.741^a^	0.626^a^	20.24^a^	68.05^a^	28.51^a^	0.396^a^	0.487^a^	0.225^a^	1.132^a^
SEm(±)	0.51	0.61	0.53	0.005	0.02	0.003	0.006	0.21	0.59	0.30	0.006	0.003	0.001	0.007
LSD (P = 0.05)	1.49	1.78	1.55	0.016	0.05	0.009	0.018	0.63	1.71	0.87	0.017	0.008	0.004	0.020

**Table 7:** Effect of different level of FYM and sources of nutrient on nutrient uptake in mustard crop (Mean data of five years).

Treatments	Uptake in seed							Uptake in stover						
	Zn (g ha^-1^)	Fe (g ha^-1^)	B (g ha^-1^)	S (kg ha^-1^)	N (kg ha^-1^)	P (kg ha^-1^)	K(kg ha^-1^)	Zn (g ha^-1^)	Fe (g ha^-1^)	B (g ha^-1^)	S (kg ha^-1^)	N (kg ha^-1^)	P (kg ha^-1^)	K(kg ha^-1^)
**A. FYM Level**														
F_0_ = control	77.84^c^	176.78^c^	54.05^c^	8.49^c^	71.16^c^	13.59^c^	9.49^c^	90.02^c^	377.9^c^	109.76^c^	16.54^c^	25.33^c^	12.18^b^	53.8^c^
F_1_ = FYM_5t_	112.98^b^	224.43^b^	80.26^b^	12.67^b^	91.27^b^	16.92^b^	12.81^b^	112.82^b^	436.23^b^	151.44^b^	21.87^b^	29.8^b^	13.62^b^	66.52^b^
F_2_ = FYM_10t_	140.68^a^	252.35^a^	128.28^a^	15.63^a^	104.75^a^	20.17^a^	17.69^a^	142.44^a^	494.56^a^	229.48^a^	25.65^a^	34.01^a^	15.61a	87.45^a^
SEm(±)	2.12	5.45	1.65	0.27	1.39	0.16	0.13	2.02	9.29	3.78	0.46	0.33	0.20	0.61
LSD (P = 0.05)	8.34	21.42	6.49	1.07	5.47	0.63	0.53	7.94	36.49	14.83	1.82	1.29	0.77	2.41
**B. Sources of Nutrient**														
M_1_ = control	66.33^e^	152.07^e^	48.92^e^	5.63^e^	60.09^e^	11.75^e^	7.69^e^	75.47^e^	329.04^e^	108.7e	12.37^e^	21.33^e^	10.19^e^	52^e^
M_2_ = Zn_1_Fe_1_B_1_S_1_	87.07^d^	181.75^d^	65.77^d^	8.33^d^	73.65^d^	14.19^d^	10.34^d^	94.36^d^	390.88^d^	132.87d	16.71^d^	25.5^d^	12.05^d^	59.97^d^
M_3_ = Zn_2_Fe_2_B_2_S_2_	108.61^c^	218.65^c^	85.9^c^	11.62^c^	89.67^c^	16.75^c^	13.42^c^	113.82^c^	443.36^c^	162.14^c^	21.19^c^	30.18^c^	13.84^c^	68.36^c^
M_4_ = En-Zn_1_Fe_1_B_1_S_1_	133.37^b^	253.69^b^	107.13^b^	15.9^b^	104.8^b^	19.71^b^	16.21^b^	135.9^b^	496.67^b^	192.34b	26.04^b^	34.23^b^	15.76^b^	78.23^b^
M_5_ = En-Zn_2_Fe_2_B_2_S_2_	157.12^a^	283.1^a^	129.93^a^	19.84^a^	117.09^a^	22.06^a^	18.99^a^	155.91^a^	521.21^a^	221.75^a^	30.45^a^	37.33^a^	17.18^a^	87.74^a^
SEm(±)	2.41	3.49	1.27	0.26	1.32	0.26	0.32	1.88	6.23	2.27	0.54	0.36	0.18	1.06
LSD (P = 0.05)	7.02	10.18	3.70	0.75	3.86	0.77	0.93	5.49	18.20	6.63	1.57	1.06	0.54	3.08

The enriched FYM with higher levels of S, Zn, Fe, and B nutrients showed its superiority not only in the removal of S, Zn, Fe and B but also N, P, K from the soil, followed by straight application. The overall mean improvement in N, P, K, S, Zn, Fe, and B uptake by mustard seed due to FYM enrichment with these nutrient at the rate of 5.0 kg Zn + 2 kg B + 10 kg Fe + 20 kg S enriched FYM @ 500 kg ha^-1^(Zn_2_B_2_Fe_2_S_2_ En) was increased by 30.57, 31.70, 41.50, 70.74, 44.66, 29.47, and 51.25% over the respective control (Zn_2_B_2_Fe_2_S_2_). The enriched treatments exhibited primarily increased efficiency of all nutrients (Table 7). This could be attributable to the beneficial influence of released organic acids in mobilising native soil nutrients, hence making them accessible to roots, as described by Sharma and Misra [59]. Due to its beneficial effects on the physical and biological features of the soil, conjunctive use of nutrients with organics was found to improve nutrient use efficiency [60]. In the present investigation, the enriched FYM, in addition to adding S, Zn, Fe, and B to the nutrient-deficient soil in naturally chelated form to provide better nutrition over longer times that gave better yields, had the beneficial effect of mobilising the native nutrients to increase their availability, which in turn caused higher nutrient use efficiency. The prime effect of externally added nutrients is causing higher crop growth, which in turn causes increased nutrient demands and so that greater removal as a result of balanced fertilisation, especially that of S, Zn, Fe, and B, due to enriched FYM along with NPK application [49–51].

### Dehydrogenase, aryl sulphatase and alkaline phosphatase activity in soil

**Fig 4:**
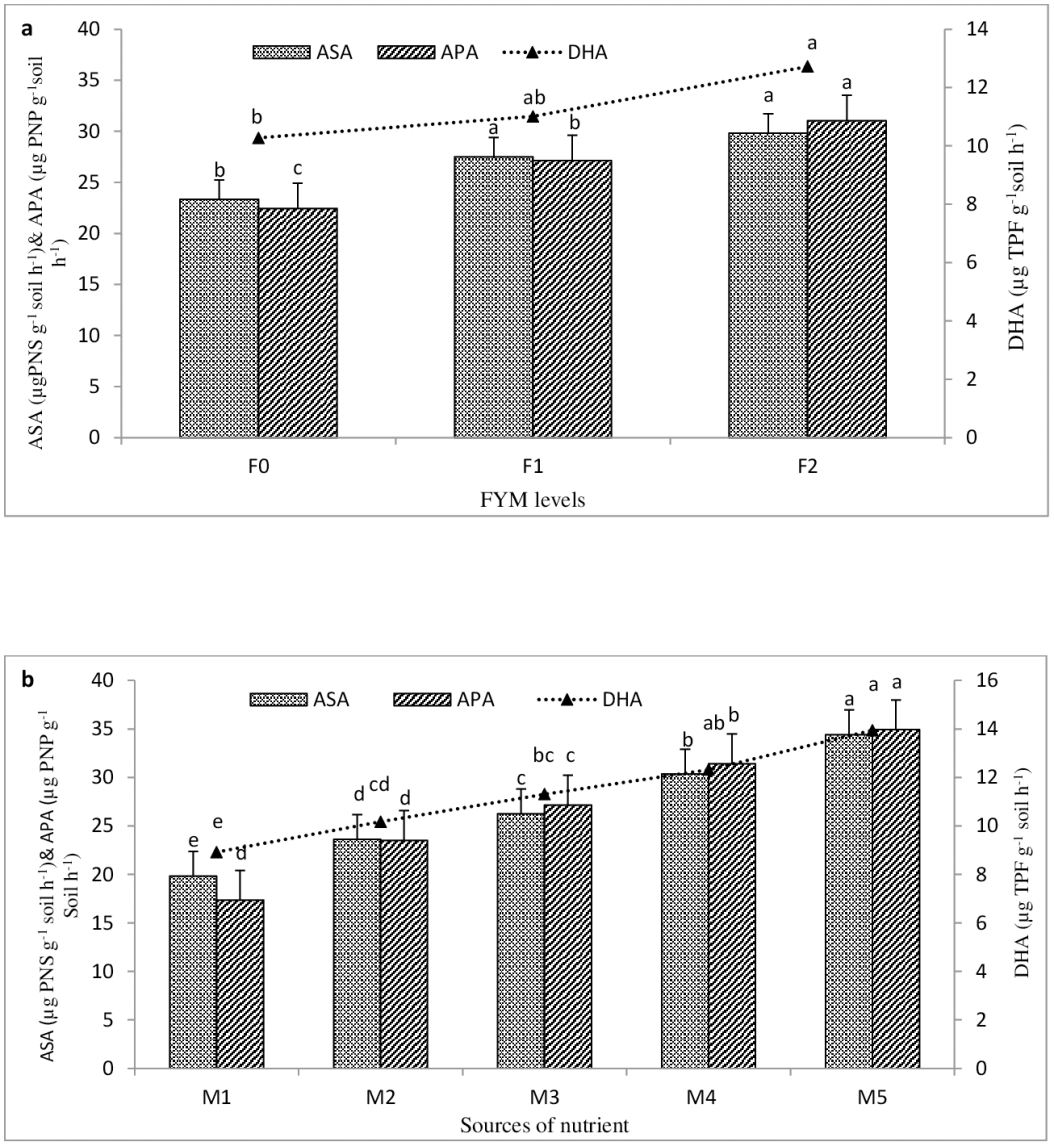
Effect of different levels of FYM (a) and sources of nutrient (b) on dehydrogenase enzyme activity, alkaline phosphatase activity and aryl sulphatase activity in soil.

The activities of alkaline phosphatase (APA), aryl sulphatase, and dehydrogenase (DHA) were significantly enhanced (p = 0.05) with elevated levels of FYM compared to the control (Fig 4a).Dehydrogenase (DHA), alkaline phosphatase (APA), and aryl sulphatase activities were raised by 23.85%, 38.32%, and 27.76% over control when FYM was used at a rate of 10 t ha^-1^. Likewise, enriching FYM with higher levels of S, Zn, Fe, and B nutrients demonstrated its superiority on dehydrogenase (DHA), alkaline phosphatase (APA), and aryl sulphatase activities when compared to their straight application (Fig 4b). Alkaline phosphatase (APA), aryl sulphatase, and dehydrogenase (DHA) showed increases in their overall mean activity by 23.25, 28.65, and 31.08%, respectively, when applied FYM was enriched at a rate of 5.0 kg Zn + 2 kg B + 10 kg Fe + 20 kg S @ 500 kg ha^-1^ (Zn_2_B_2_Fe_2_S_2_ En) over respective straight application.

Likewise, the activities of dehydrogenase, alkaline phosphatase, and aryl sulphatase were raised by 21.14, 33.58, and 28.49% under the use of M_4_ treatment (Zn_1_B_1_Fe_1_S_1_ En) compared to the corresponding straight application (Zn_1_B_1_Fe_1_S_1_) of micro- and secondary nutrients. These findings revealed that under enriched treatment of FYM, highest dehydrogenase (DHA) and aryl sulphatase activities were reported under the M_5_ (Zn_2_B_2_Fe_2_S_2_ En) treatment. However, maximum alkaline phosphatase (APA) activity was observed under the treatment of M_4_ (Zn_1_B_1_Fe_1_S_1_ En). According to Speir [61], soil enzyme activity rises as soil organic matter (SOM) levels increase in the field. An increase in the amount of organic matter in the soil was associated with an increase in enzymatic activities and low molecular organic acids [62]. There has been prior research connecting elevated enzymatic activity with increased microbial biomass brought on by soil enrichment with organic matter. Because the enzyme fraction was preserved as the amount of humus in the soil grew, Martens et al. [63] also observed increases in DHA and APA. The current study confirmed the earlier findings by demonstrating a substantial positive correlation between SOC, microbial biomass, and different enzyme activity. Tejada et al. [54], Kumar et al. [64] Okur et al. [65] and Wu et al. [53] have drawn similar results in previous studies.

### Nutrient efficiency indices and economic outcomes of Indian mustard

Nutrient efficiency indices demonstrate how the greater doses of FYM applied significantly enhanced the nutrient mobilisation indices (MEI) of N, P, K, S, Zn, Fe, and B (Table 8). The highest nutrient mobilisation indices (MEI), which were considerably better than the control, were seen when FYM was used at a rate of 10 tons per hectare. The ME results showed that, in comparison to stover, applying larger quantities of FYM led to more nutrient translocation towards the seed. The increased nutrient translocation in stovers relative to seeds was indicated by the lower MEI value. Moreover, it has been noted that mustard seeds have a higher nutrient content than stover, with the exception of K content, which is higher in stover. Accordingly, the current study also determined the lowest value of K’s nutrient mobilisation indices (MEI). Additionally, it was observed that the application of fortified FYM, including S, Zn, Fe, and B, improved the mobilisation efficiency of N, P, and K over control in addition to increasing the nutrient mobilisation indices (MEI) of Zn, Fe, B, and S. Higher efficiency of micronutrients was caused by the enriched FYM, primarily because it helped mobilise native nutrients and make them more available. Additionally, it added S, Zn, Fe, and B to the soil in naturally chelated form, which improved nutrition over a longer period of time and increased yields. Over time, the macronutrient fertiliser’s partial factor productivity (NPK) has decreased. A contributing factor to this decrease is the growing micronutrient deficiency in soil. Numerous studies have determined that micronutrients, albeit being required in trace amounts, contribute significantly to the improvement of NPK’s utilisation efficiency [66].

**Table 8:** Effect of different level of FYM and sources of nutrient on nutrient Mobilization efficiency index (%) in mustard crop (Mean data of five years).

Treatments	Mobilization efficiency index (%)						
	S	B	Zn	Fe	N	P	K
**A. FYM Level**							
F_0_ = control	1.33^a^	1.25^a^	2.3^a^	1.24^a^	7.46^a^	2.97^a^	0.46^a^
F_1_ = FYM_5t_	1.4^a^	1.3^a^	2.47^a^	1.28^a^	7.62^a^	3.1^a^	0.47^a^
F_2_ = FYM_10t_	1.48^a^	1.38^a^	2.46^a^	1.27^a^	7.69^a^	3.23^a^	0.50^a^
SEm(±)	0.01	0.02	0.02	0.01	0.02	0.05	0.003
LSD (P = 0.05)	0.05	0.06	0.06	0.04	0.07	0.18	0.01
**B. Sources of Nutrient**							
M_1_ = control	1.14^c^	1.06^b^	2.22^b^	1.17^a^	7.09^c^	2.9^b^	0.37^a^
M_2_ = Zn_1_Fe_1_B_1_S_1_	1.26^bc^	1.24^ab^	2.34^ab^	1.19^a^	7.36^bc^	2.99^ab^	0.44^a^
M_3_ = Zn_2_Fe_2_B_2_S_2_	1.38^abc^	1.33^ab^	2.42^ab^	1.26^a^	7.59^abc^	3.09^ab^	0.5^a^
M_4_ = En-Zn_1_Fe_1_B_1_S_1_	1.56^ab^	1.42^a^	2.5^a^	1.31^a^	7.87^ab^	3.21^ab^	0.53^a^
M_5_ = En-Zn_2_Fe_2_B_2_S_2_	1.67^a^	1.5^a^	2.57^a^	1.4^a^	8.06^a^	3.3^a^	0.55^a^
SEm(±)	0.02	0.02	0.01	0.01	0.06	0.02	0.01
LSD (P = 0.05)	0.07	0.05	0.04	0.03	0.16	0.05	0.02

**Fig 5:**
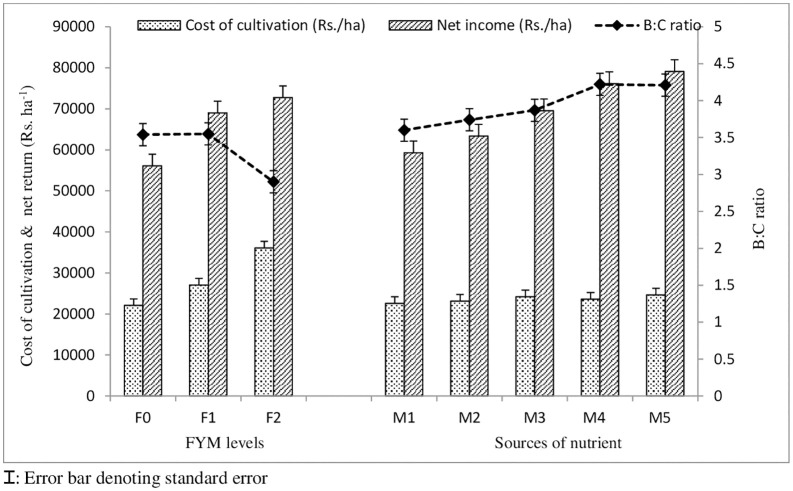
Economics of the treatments influenced by different level of FYM and nutrient enriched organic sources.

In terms of economic analysis, the results showed that among the main plot treatments of FYM (control (F_0_), FYM @ 5 t ha^-1^ (F_1_), and FYM @ 10 t ha^-1^ (F_2_), the highest cultivation cost was calculated in treatment F_2_, whereas the lowest cultivation cost was found in treatment F_0_. In a similar manner, treatment F_2_ yielded the highest net return value, followed by treatment F_1_. But the benefit to cost ratio was highest in F_1_ followed by F_2_, and lowest in the control. Economic analysis of the treatments revealed that while there was considerable improvement in the B:C ratio up to 3.55 when FYM levels were increased from control to 5 t ha^-1^, the B:C ratio decreased by 18.07% over control when FYM levels were further enhanced to 10 t ha^-1^. Additionally, it was observed that FYM enriched with micro- and secondary nutrients (Zn, Fe, B, and S) improved the net return and had a positive effect on the B:C ratio compared to the control. The B:C ratio was likewise raised by the direct (non-fortified) application of Zn, Fe, B, and S, although the percentage improvement was lower than when they were applied through the fortified FYM. The highest B:C ratio (4.22) was recorded in the nutrient-fortified FYM treatment of M_4_ (2.5 kg Zn + 1 kg B + 5 kg Fe + 10 kg S En- FYM @ 500 kg h^-1^). The lowest ratio was measured in the control (Fig 5).

Fortifying FYM with micro and secondary nutrients (S, Zn, Fe, and B) at a rate of 2.5 kg Zn + 1 kg B + 5 kg Fe + 10 kg S enriched FYM @ 500 kg ha^-1^ improved the B:C ratio by 17.22% over control, and under M_5_ treatment (5 kg Zn + 2 kg B + 10 kg Fe + 20 kg S enriched FYM @ 500 kg h-1), the improvement was 16.94%. Results of the experiment indicated that the economically maximum yield of 28.72 q/ha with a B:C ratio of 4.22 was generated by applying fortified FYM enriched with micro and secondary nutrients at a rate of 2.5 kg Zn + 1 kg B + 5 kg Fe + 10 kg S enriched FYM @ 500 kg ha^-1^. The delivery of an appropriate amount of micro and secondary nutrients (treatment M_4_) through 500 kg FYM was found to enhance mustard yield and increase farmers’ economic return. These kinds of studies are crucial to improving mustard yields in Indian border regions that experience abiotic salinity stress.

## Conclusion

In addition to increasing cropping intensity, the majority of Indian soils lack micro- and secondary nutrients, specifically zinc, iron, sulphur, and B, as a result of the usage of fertilisers devoid of micronutrients and the limited addition of organic manures.. Micronutrient deficiencies in Indian soils have caused a decrease in the production and nutrient content of certain oilseed crops. Furthermore, abiotic variables like soil salinity and sodicity, which also lower the availability of micronutrients like zinc (Zn), iron (Fe), boron (B), and sulphur (S) in the soil, are mostly to blame for the low crop yield in the mustard-growing region.. Consequently, prudent plant nutrition management in these soils is equally as important as soil reclamation. In these arid and semi-arid areas that are also getting irrigation with saline water, micronutrient-enriched FYM could be a helpful strategy for alleviating nutrient deficiencies. By keeping minerals in their naturally chelated state, nutrient-fortified FYM helps to improve nutrition over time and boost yields. The findings demonstrated that when fortified FYM containing zinc (Zn), iron (Fe), boron (B), and sulphur (S) was applied at a rate of 2.5 kg Zn + 1 kg B + 5 kg Fe + 10 kg S enriched FYM @ 500 kg ha^-1^, the highest B:C ratio (4.22) was reached with a seed yield of 28.72 q ha^-1^. The availability of S, Zn, Fe, and boron, as well as the MBC, SOC, and MBN, were significantly higher in FYM that was fortified with micro- and secondary nutrients than in the control. Alkaline phosphatase, aryl sulphatase, and dehydrogenase activity in soil were also markedly increased by nutrient fortification. Additionally, the single practice of FYM fortification with secondary and micronutrients (Zn_1_B_1_Fe_1_S_1_En) boosted stover and seed yield by 16.44% and 15.57 per cent, respectively, compared to straight application (Zn_1_Fe_1_B_1_S_1_). In the dry and semi-arid northwest of India, in the states of Rajasthan, Gujarat, Haryana, and portions of Uttar Pradesh and Madhya Pradesh, this research may provide a useful technique for raising mustard production.
